# A Novel Approach for Glycero-(9,10-trioxolane)-Trialeate Incorporation into Poly(lactic acid)/Poly(ɛ-caprolactone) Blends for Biomedicine and Packaging

**DOI:** 10.3390/polym16010128

**Published:** 2023-12-30

**Authors:** Olga V. Alexeeva, Anatoliy A. Olkhov, Marina L. Konstantinova, Vyacheslav V. Podmasterev, Tuyara V. Petrova, Levon Yu. Martirosyan, Olga K. Karyagina, Sergey S. Kozlov, Sergey M. Lomakin, Ilya V. Tretyakov, Valentina Siracusa, Alexey L. Iordanskii

**Affiliations:** 1 Emanuel Institute of Biochemical Physics of Russian Academy of Sciences, 119334 Moscow, Russia; aolkhov72@yandex.ru (A.A.O.); kisisinova@yandex.ru (M.L.K.); vpodmasterev@yandex.ru (V.V.P.); levon-agro@mail.ru (L.Y.M.); olgakar07@mail.ru (O.K.K.); sergeykozlov1@gmail.com (S.S.K.); lomakin@sky.chph.ras.ru (S.M.L.); 2 N.N. Semenov Federal Research Center for Chemical Physics Russian Academy of Sciences, 119991 Moscow, Russia; tuyara.2312@mail.ru (T.V.P.); tretiakovi.v@yandex.ru (I.V.T.); aljordan08@gmail.com (A.L.I.); 3 Academic Department of Innovational Materials and Technologies Chemistry, Plekhanov Russian University of Economics, 117997 Moscow, Russia; 4 Department of Chemical Science (DSC), University of Catania, Viale A. Doria 6, 95125 Catania, Italy

**Keywords:** polylactic acid, polycaprolactone, PLA/PCL blend films, ozonide, antibacterial activity

## Abstract

The product of ozonolysis, glycero-(9,10-trioxolane)-trioleate (ozonide of oleic acid triglyceride, [OTOA]), was incorporated into polylactic acid/polycaprolactone (PLA/PCL) blend films in the amount of 1, 5, 10, 20, 30 and 40% *w*/*w*. The morphological, mechanical, thermal and antibacterial properties of the biodegradable PLA/PCL films after the OTOA addition were studied. According to DSC and XRD data, the degree of crystallinity of the PLA/PCL + OTOA films showed a general decreasing trend with an increase in OTOA content. Thus, a significant decrease from 34.0% for the reference PLA/PCL film to 15.7% for the PLA/PCL + 40% OTOA film was established using DSC. Observed results could be explained by the plasticizing effect of OTOA. On the other hand, the PLA/PCL film with 20% OTOA does not follow this trend, showing an increase in crystallinity both via DSC (20.3%) and XRD (34.6%). OTOA molecules, acting as a plasticizer, reduce the entropic barrier for nuclei formation, leading to large number of PLA spherulites in the plasticized PLA/PCL matrix. In addition, OTOA molecules could decrease the local melt viscosity at the vicinity of the growing lamellae, leading to faster crystal growth. Morphological analysis showed that the structure of the films with an OTOA concentration above 20% drastically changed. Specifically, an interface between the PLA/PCL matrix and OTOA was formed, thereby forming a capsule with the embedded antibacterial agent. The moisture permeability of the resulting PLA/PCL + OTOA films decreased due to the formation of uniformly distributed hydrophobic amorphous zones that prevented water penetration. This architecture affects the tensile characteristics of the films: strength decreases to 5.6 MPa, elastic modulus E by 40%. The behavior of film elasticity is associated with the redistribution of amorphous regions in the matrix. Additionally, PLA/PCL + OTOA films with 20, 30 and 40% of OTOA showed good antibacterial properties on *Pseudomonas aeruginosa*, *Raoultella terrigena* (*Klebsiella terrigena*) and *Agrobacterium tumefaciens*, making the developed films potentially promising materials for wound-dressing applications.

## 1. Introduction

Biodegradable polymers are of great technological interest as a replacement for hydrocarbon polymers for packaging, for biomedical applications such as tissue engineering, drug delivery and powder coating, and in agriculture [1,2,3]. Recently, there has been an increasing proportion of research focusing on aliphatic polyesters, including polylactides (PLAs) and poly-ε-caprolactones (PCLs), as the most important biodegradable materials [4]. Polylactic acid (PLA) is one of the most promising polymers for biomedical applications due to its high strength characteristics, ideal biocompatibility and complete biodegradability. It is actively used for bone tissue therapy and tissue engineering [5,6]. PLA is a biocompatible polymer obtained from the fermentation of carbohydrates, allowing its large-scale production with lower economic costs and low greenhouse gas emissions [7].

However, the use of pure PLA is limited by its brittleness. An effective method for increasing the toughness of brittle polymers is to blend them with elastomers [8]. Various studies have shown that PLA can be modified using various synthetic, semi-synthetic or natural polymers to improve its properties due to a synergistic effect. One possible blending agent for PLA is the biocompatible polyester PCL [9].

Poly(ε-caprolactone) (PCL) is a hydrophobic semicrystalline aliphatic polyester that is synthesized by ring-opening polymerization from a caprolactone monomer initiated by divalent alcohols and tin (II) or tin salts [10]. PCL has high-mobility chain segments with low intermolecular interactions, resulting in a very low melting point (+60 °C) and glass transition temperature (−60 °C). The structure of PCL is a combination of ordered crystalline and disordered domains, which improves elasticity. Due to its high flexibility, PCL exhibits high elongation at break and low tensile strength. PCL is commonly used for drug delivery systems, microporous foams and long-term implants. PCLs with lower molecular weight are used as plasticizers or compatibilizers and adhesives for other biopolymers [11]. PLA/PCL binary mixtures generally have improved physicochemical properties and biodegradability. Amorphous PLA has a high degradation rate and provides tensile strength, while PCL improves toughness but has a much lower degradation rate [12,13]. A decrease in vapor permeability has been shown when adding PLA to PCL. According to the results of numerous studies, the best mixing and synergistic properties in PCL and PLA blends are provided with an 80/20 PLA/PCL ratio [14]. The use of PLA/PCL mixtures is relevant as promising materials for tissue engineering and bone anchorage devices, as well as for controlled drug release and biodegradable packaging. 

Currently, more and more modern, high-tech wound dressings are being offered, which are gradually replacing gauze dressings in medicine [15,16]. Biopolymer films already available on the market provide an effective barrier to external contamination and bacteria, whereas the water vapor transport properties are not usually taken into account. State-of-the art polymer materials suitable for wound dressing should provide good vapor permeability, but on the other hand, it should not be high enough to balance a moist environment on the surface of the wound by reducing the loss of water vapor from open tissues. Under these conditions, the formation of a scab in shallow wounds is prevented and accelerated regeneration of the epidermis occurs. Sometimes these films contain an antibacterial component, such as the widely used chlorhexidine.

In this work, we consider the introduction of a strong antibacterial agent, OTOA, into a PLA/PCL blend film. OTOA is non-toxic, biocompatible, has good biodegradability and antimicrobial activity. In recent studies [17,18], it has been already shown that the introduction of OTOA into PLA fibers and films led to improvement in the physicochemical properties of the material. We have now obtained novel film materials based on a PLA/PCL blend with the addition of various amounts of OTOA (0–40 wt %) as a modifier. PLA/PCL films with different OTOA contents were studied using DSC, XRD and FT-IR spectroscopy. The morphological, physicochemical and mechanical properties, vapor permeability and water absorption of the developed films were studied. The influence of the amount of introduced OTOA on the physicochemical and functional properties of the blended film materials has been established, which gives some insight into the peculiarities of the interaction of PLA/PCL with OTOA. The antibacterial activity of PLA/PCL + OTOA films on bacteria that are highly resistant to antibiotics, such as *Pseudomonas aeruginosa*, *Raoultella terrigena* (*Klebsiella terrigena*) and *Agrobacterium tumefaciens*, was also investigated.

## 2. Materials and Methods

### 2.1. Materials

**Figure 1:**
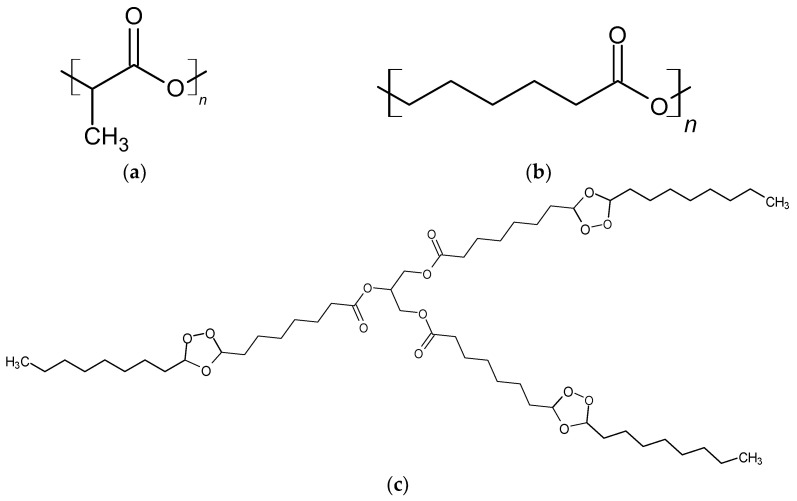
Chemical structure of PLA (**a**), PCL (**b**) and OTOA (**c**).

To obtain film materials, NatureWorks^®^ Ingeo™ 3801X Injection Grade PLA (SONGHAN Plastics Technology Co., Ltd., Shanghai, China) with a viscosity-average molecular weight of 1.9 × 10^5^ g/mol was used; PCL was used (SONGHAN Plastics Technology Co., Ltd., Shanghai, China) with a molecular weight of 90,000 (Figure 1). Solutions were prepared using ≥99.5% dry purified chloroform (Sigma-Aldrich Inc., St. Louis, MO, USA); Glycero-(9,10-trioxolane)-trioleate (oleic acid triglyceride ozonide (OTOA)) was provided by Medozon (Moscow, Russia), and its chemical structure was described previously [17,18]. All reagents were used as received.

### 2.2. Preparation of Films

All film materials used in this study were prepared by solvent evaporation from the corresponding chloroform solutions. For the reference film, PLA/PCL blend (80/20 *w*/*w*%, 1 g total) was dissolved in 50 mL of chloroform. For the PLA/PCL + OTOA films, the corresponding amounts of OTOA (1, 3, 5, 10, 20, 30, 40 wt %) were added to the PLA/PCL 80/20 *w*/*w*% blend, so that the total amount of solutes was 1 g, which was followed by dissolution in 50 mL of chloroform. Obtained solutions were continuously stirred for 12 h, poured into Petri dishes (10 cm diameter) and dried to constant weight at room temperature. All films were obtained in the same ambient conditions (T = 22 °C) in order to ensure the equal rate of chloroform evaporation. Films of pure PLA and PCL were obtained in the same way as described above.

### 2.3. IR Spectroscopy

FTIR spectra of PLA/PCL blend films were recorded with a NETZSCH TG 209 F1 Libra thermoanalytical balance (NETZSCH-Gerätebau GmbH, Selb, Germany) and a Bruker Tensor 27 IR Fourier spectrometer (Bruker Corporation, Billerica, MA, USA), combined with a PIKE MIRacle™ accessory (PIKE Technologies, Madison, WI, USA). This attachment is equipped with a Teflon cell with Ge crystal and ATR plates, which allows the measurements of solid samples. The samples were tightly clamped to the surface of the Ge crystal to ensure optical contact. IR spectra were recorded in the range of 4000–400 cm^−1^ with a resolution of 4 cm^−1^ and averaged over 16 consecutive scans.

### 2.4. DSC

The thermophysical characteristics of the PLA/PCL + OTOA compositions were measured on a DSC 204 F1 Phoenix^®^ (NETZSCH-Gerätebau GmbH, Selb, Germany) differential scanning calorimeter with a heating rate of 10 K/min in an inert Ar atmosphere in the temperature range of 25–200 °C. The DSC experiments included only one stage of heating due to the high degree of exothermic decomposition of glycero-(9,10-trioxolane)-trioleate (OTOA) and subsequent volatilization of its decomposition products. On the other hand, we wanted to characterize the morphology of the originally obtained compositions, rather than “erasing their thermal memory” or thermodynamically “balancing” their primary structure. The degree of crystallinity (*χ*) of PLA in PLA/PCL (80:20) + OTOA composites was calculated according to the equation

(1) $$\chi =\frac{\Delta H_{m}-\Delta H_{cc}}{\Delta H_{m}^{100}\times (1-\beta -\gamma )}\times 100\%$$

where Δ*H_m_*—the experimental enthalpy of melting, Δ*H_m_*^100^—the theoretical melting enthalpy value of the 100%-crystalline poly(L-lactide), which is 93.2 J/g [19], *γ*—mass fraction of glycero-(9,10-trioxolane)-trioleate ozonide (OTOA) additive and *β*—mass fraction of PCL. Table 1 presents the percent concentrations and the mass fraction values of studied PLA/PCL + OTOA compositions.

Deconvolution of complex DSC peaks was performed using NETZSCH Peak Separation 2006.01 program (NETZSCH-Gerätebau GmbH, Selb, Germany) employing the Fraser-Suzuki algorithm for asymmetric DSC curves [17,20,21]. 

### 2.5. X-ray Diffraction Analysis

XRD patterns of the PLA/PCL blend films were obtained using DRON-3M X-ray diffractometer (Bourevestnik, St. Petersburg, Russia) as described previously [17]. Relative crystallinity was estimated as

χ = I_C_/(I_C_ + I_A_), (2)

where I_C_ and I_A_ are the integral intensities corresponding to the respective crystalline and amorphous phases [22,23].

### 2.6. Morphology

#### 2.6.1. Optical Microscopy

Surface morphology of PLA/PCL films with different OTOA contents was studied using an Olympus CX21 microscope (Olympus Corp., Tokyo, Japan) equipped with a digital camera. Image capture and processing was performed using MICAM 3.02 software.

#### 2.6.2. Sorption Capacity

To determine water sorption, PLA/PCL film samples were cut into strips of 4 × 4 cm^2^ and kept for 24 h under standard atmospheric conditions (44 ± 2% relative humidity and 20 ± 2 °C). After exposure, the mass of the samples (m_1_) was measured, and then they were immersed in distilled water for 24 h [24]. The removed wet samples were hung in the open air for 30 min to remove excess water from the surface of the samples. The mass (m_2_) of the wet samples was then measured. Water sorption was calculated using the formula:

%Q = (m_2_ − m_1_)/m_1_ × 100% (3)

where m_1_ and m_2_ are the mass of the sample before and after immersion in water. By measuring the contact angle in a semi-angle modification [25] for a drop of water placed on the surface, the degree of hydrophilicity of PLA/PCL blended films was determined. Drops sizes were determined using an OLIMPUS CX21 optical microscope (Olympus Corp., Tokyo, Japan) and then processed using MICAM 3.02 software.

#### 2.6.3. Moisture Permeability of Films by Moisture Permeable Cup Method

Vapor permeability measurements were carried out using the standard ASTM E96 method [26,27,28]. A sample of the film covers the neck of the cup, inside of which there is distilled water, which creates water vapor pressure inside the cup. The installation is placed in a desiccator with silica gel at a constant temperature. Weight loss is recorded as a function of time. The slope is evaluated by linear regression (R^2^ > 0.99). The water vapor transmission rate (*WVTR*), water vapor permeance, and water vapor permeability (*WVP*) are calculated according to Equations (4)–(6) [29,30,31]:

(4) $$WVTR\left(\frac{g}{d\cdot m^{2}}\right)=\frac{∆\omega }{∆t\cdot A}$$

(5) $$permeance\left(\frac{g}{d\cdot m^{2\;}\cdot Pa}\right)=\frac{WVTR}{∆P}$$

(6) $$WVP\left(\frac{g}{d\cdot m\cdot Pa}\right)=permeance\;\cdot thickness$$

where Δ*ω*/Δ*t* (g/d) is the weight loss of the water in the cup per unit of time, *A* (m^2^) is the actual area of the cup neck and Δ*P* (Pa) is the water vapor pressure differential calculated as 3.5670 kPa at 30 °C, assuming full water vapor saturation of the cup headspace and dried environment provided by the silica gel. Each test was performed in triplicate.

### 2.7. Mechanical Properties

Mechanical properties of the samples were studied on a Zwick Z010 instrument (ZwickRoell GmbH & Co., Ulm, Germany) at room temperature. The shape of testing samples cut from polymer-blended PLA/PCL films was given in [17]. The loading speed was 1 mm/min during measurements. Sample loading diagrams were obtained (load F versus deformation ԑ). From the loading diagrams, the elastic modulus E, tensile strength σ and relative elongation at break (rupture) were determined. Five samples of each PLA/PCL + OTOA film were taken for mechanical tests. Results are presented as mean ± standard deviation at a significance level of *p* < 0.05.

### 2.8. Measurement of Antibacterial Activity

For experiments, *Pseudomonas aeruginosa* (Korea Type Culture Collection), *Raoultella terrigena* (*Klebsiella terrigena*) and *Agrobacterium tumefaciens* strains from the Korean Cell Line Bank were used. After adding them to TSA (tryptic soy agar) medium, the culture was enriched for 18–24 h at a temperature of 35–37 °C.

The antibacterial effectiveness of PLA/PCL + OTOA film samples was measured by the Murray paper disk method [32,33]. The strain in the main culture was determined by increasing the activity of trypticase soy broth (TSB) under optimal conditions for approximately 6 h. Then, 100 μL of the culture medium of each strain was evenly spread on TSA (tryptic soy agar). PLA/PCL films with 10%, 20%, 30% and 40% OTOA concentrations were used for antibacterial activity tests.

The inoculum was prepared from an 18–20 h agar culture in meat-peptone broth, adjusting the turbidity to 0.5 McFarland standard. The resulting broth culture was diluted 10 times with a sterile isotonic sodium chloride solution, which corresponded to a final concentration of 1 × 10^5^ CFU/mL. Sterile cotton swabs were used to apply the inoculum to Petri dishes with a dense nutrient medium. The swab was immersed in a suspension of microorganisms. Excess moisture was removed by squeezing the swab against the wall of the tube. Inoculation onto the surface of the agar medium was carried out using streak movements, periodically rotating the Petri dish by 60°.

Disks made of PLA/PCL + OTOA films with a diameter of 6.0 ± 0.1 mm were applied to the seeded surface using sterile tweezers. The concentration of the active fraction per disk fluctuated depending on the experiment from 20 to 40%. Disks of the reference PLA/PCL film and 100% OTOA film were used as comparison objects. After applying the disks, the dishes were placed in a thermostat and incubated for 18–20 h at 37 °C. At the end of incubation, the growth zone retention was calculated. When measuring the zones, we were guided by complete inhibition of visible growth [34,35]. The antibacterial activity was measured, and the experiment was triplicated.

## 3. Results and Discussion

### 3.1. FTIR Spectroscopy

**Figure 2:**
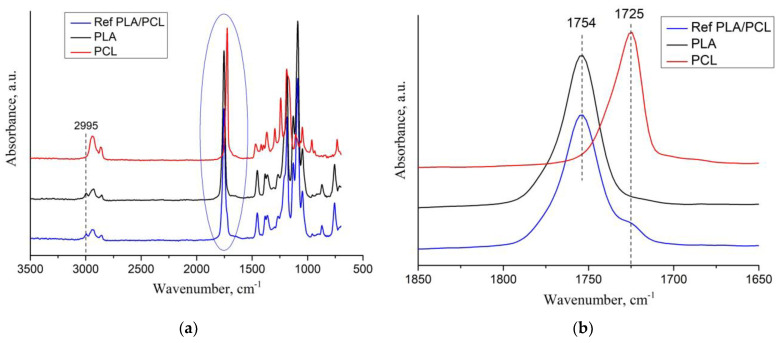
FTIR spectra of pristine PLA, PCL and PLA/PCL films (**a**), close-up view of FTIR spectra at 1650–1850 cm^−1^ interval (**b**).

**Figure 3:**
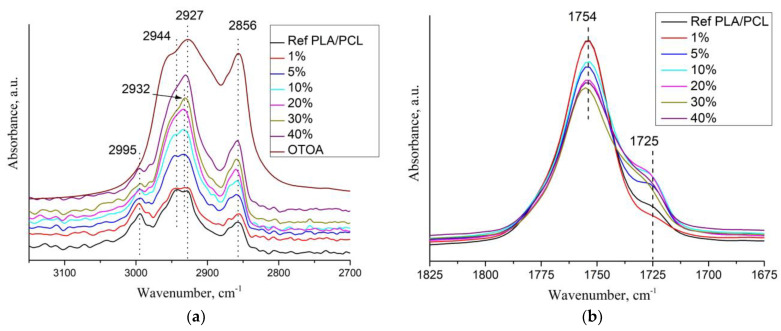
FTIR spectra of pristine PLA/PCL film, PLA/PCL + OTOA films and pure OTOA: (**a**) Close-up view of FTIR spectra at 2700–3200 cm^−1^ interval, (**b**) FTIR spectra at 1675–1825 cm^−1^ wavenumber region.

To clarify the features of the chemical interaction between PLA, PCL and OTOA, FTIR spectra of PLA/PCL (Figure 2) and PLA/PCL + OTOA films were obtained (Figure 3).

Figure 2a shows the Fourier transform infrared (FTIR) spectra of the PLA, PCL and PLA/PCL film samples. The PLA film spectrum showed characteristic bands at 1362 cm^−1^, 1455 cm^−1^ and 1754 cm^−1^, resulting from -CH_3_ bending and C=O stretching vibrations, respectively. In addition, strong C-O stretching bands were observed in the 1000–1200 cm^−1^ range, as well as a small peak at 2995 cm^−1^ attributed to the asymmetric stretching of the –CH group [36]. The FTIR spectrum for the pristine PCL film showed characteristic bands at 1295 cm^−1^ (C-C stretching in crystalline phase), 1242 cm^−1^ and 1170 cm^−1^ (asymmetric and symmetric C-O-C stretching) [37]. A characteristic peak at 1725 cm^−1^ corresponded to carbonyl group stretching, while 2856 and 2940 cm^−1^ bands could be related to symmetric and asymmetric -CH_2_ stretching [17]. The FTIR spectrum for the PLA/PCL film showed that the typical PCL bands at 1170–1190 cm^−1^ and 1294 cm^−1^ disappeared, overlapping with stronger PLA characteristic bands, while small shoulder peaks existed at 1242 and 1294 cm^−1^. In addition, a shoulder at 1725 cm^−1^ was visible in the FTIR spectrum for the PLA/PCL film, which was related to the stretching of the C=O groups in PCL (Figure 2b). Therefore, the FTIR spectrum of the PLA/PCL film represents the mixture of CH_3_, C=O and C–O bands being characteristic of PLA, with the presence of small C=O and CH_2_ peaks reflecting the 20% PCL content in the PLA/PCL film. Moreover, the absence of a characteristic band at 1294 cm^−1^ is evidence that the most of the PCL in the composite PLA/PCL film is amorphous.

The addition of OTOA reduced the intensity of the C-O, C=O and CH_3_ bands in the FTIR spectra of the PLA/PCL blends compared to pure PLA/PCL and PLA/PCL + 1% OTOA (Figure 3b). This decrease correlated with the increase in the additive concentration. This occurred because the C=O group in PLA interacted through hydrogen bonding with the –OH group of PCL and OTOA, which was described previously [17].

The absorption bands at 2944 and 2995 cm^−1^ are attributed to the asymmetric stretching vibrations of the –CH group of PLA (Figure 3a). As can be seen, the absorption bands of PLA/PCL and OTOA partially overlap. In the spectra of PLA/PCL + OTOA films, there were two bands at 2927 cm^−1^ and 2856 cm^−1^, which are attributed to the asymmetric and symmetric stretching vibrations of the -CH_2_ groups [38,39]. The spectra clearly showed that the intensity of these peaks was increased upon an increase in the OTOA content in the films. Since PLA does not contain -CH_2_ groups in its chemical structure, and the -CH_2_ group is present in abundance in PCL and OTOA, the observed intensity increase of 2927 cm^−1^ and 2856 cm^−1^ characteristic bands in the FTIR spectra of PLA/PCL + OTOA films is clear evidence of the inclusion of OTOA in the supramolecular structure of the PLA/PCL films. Additionally, the band at 2927 cm^−1^ showed a slight shift to higher wavenumber values (up to 2932 cm^−1^) for PLA/PCL + OTOA samples with OTOA content higher than 10%, which could indicate the interaction of OTOA molecules (mainly through the ozonide rings) with the PLA/PCL matrix [17].

Based on the FTIR spectra analysis, it can be assumed that the observed interactions between the polar groups in PLA/PCL and the ozonide rings in OTOA promote conformational changes associated with the reorientation of polar groups in PLA, which contributes to an increase in the segmental mobility of the PLA/PCL polymer chains [40]. Thus, low-molecular-weight OTOA could act as a plasticizer, affecting the mechanical and physicochemical properties of PLA film materials. 

### 3.2. Differential Scanning Calorimetry

**Figure 4:**
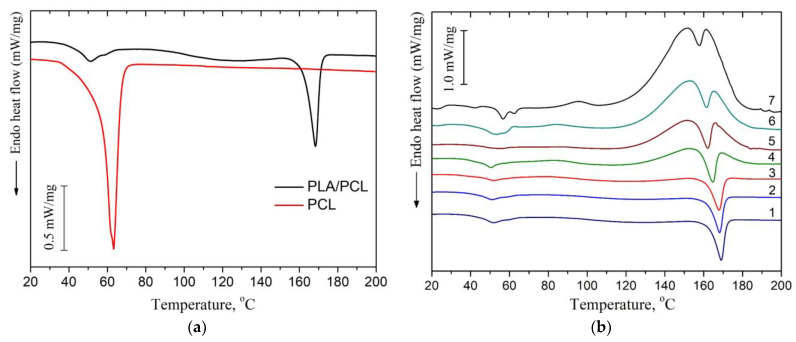
(**a**) DSC heat flow curves of PLA and PLA/PCL films; (**b**) DSC thermograms for the samples of pristine PLA/PCL (1), PLA/PCL + 1% (2), + 5% (3), + 10% (4), + 20% (5), + 30% (6), + 40% OTOA (7), respectively.

Figure 4a shows the DSC curves of the first heating for the pristine PLA/PCL and PCL films, while DSC curves for PLA/PCL + OTOA film samples are shown in Figure 4b.

The DSC thermogram of reference PLA/PCL film (Figure 4a) showed an endothermic peak at 168.4 °C, which is the typical melting point of PLA [17,40], and a double peak at 51.1 °C and 58.6 °C, which corresponds to the devitrification of PLA and melting of PCL. Neat PCL film exhibited an endothermic melting peak at 63.2 °C, which is also typical for the melting of PCL [37,41].

**Figure 5:**
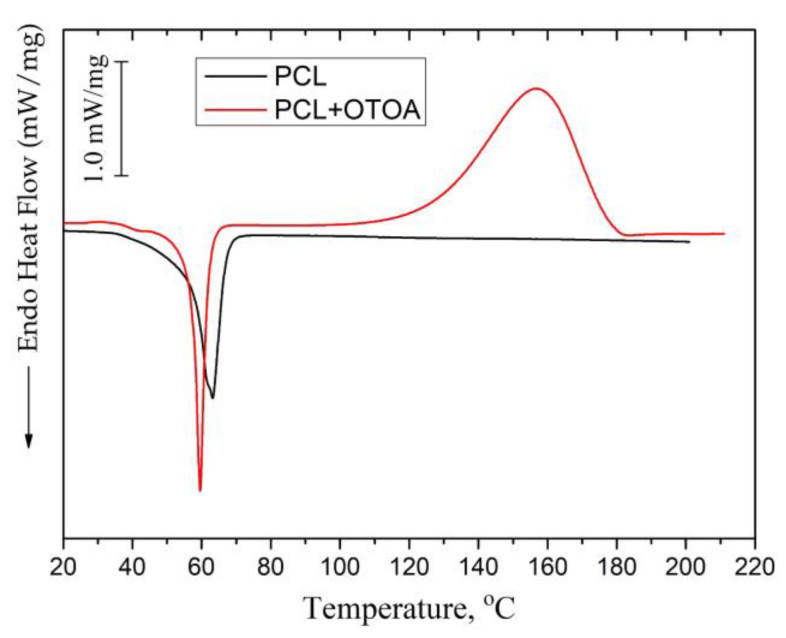
DSC heat flow curves of PCL and PCL + OTOA 35% films.

In the DSC thermograms of the first heating of PLA/PCL + OTOA films, the endothermic peak in the range of 150–180 °C corresponded to the melting of PLA (T_m_). The low-temperature transition in the interval of 40–60 °C could be attributed to the devitrification of PLA (T_g_) and melting of PCL, and the corresponding transition temperatures T_g_ and T_m_ are given in Table 2. When OTOA is added to the matrix and its mass fraction in the film increases, the temperatures of those two transitions shift to higher temperatures up to 55.1 °C and 62.5 °C, respectively, which indicates the participation of OTOA in the melting process of PCL. To test this, DSC curves for the PCL and PCL + 35% OTOA films were compared (Figure 5), and the PCL melting peak was shown to shift from 63.2 °C to 59.5 °C with simultaneous narrowing of the endothermic peak. This indicates that OTOA could act as a plasticizer for PCL and to some extent promote crystallization of PCL. This assumption was supported by the DSC data for PLA/PCL + OTOA films, for which the melting transition for PCL could be reliably differentiated only for films with 30% and 40% OTOA. For PLA/PCL film with 40% OTOA, a double PCL melting peak could be observed around 60 °C in the DSC curve. It could be concluded that PCL in the PLA/PCL + OTOA films with less than 30% OTOA is generally in the amorphous state, as was previously proposed based on FTIR data. An increase in OTOA content above 30% promotes additional PCL crystallization, which manifests in corresponding DSC curves and will be addressed further by XRD. Additionally, small cold-crystallization exothermic peaks of PLA were observed in DSC thermograms for PLA/PCL + OTOA films with an OTOA content higher than 5%. The corresponding values for the temperature (T_cc_) and enthalpy (ΔH_cc_) of PLA cold crystallization are given in Table 2.

**Figure 6:**
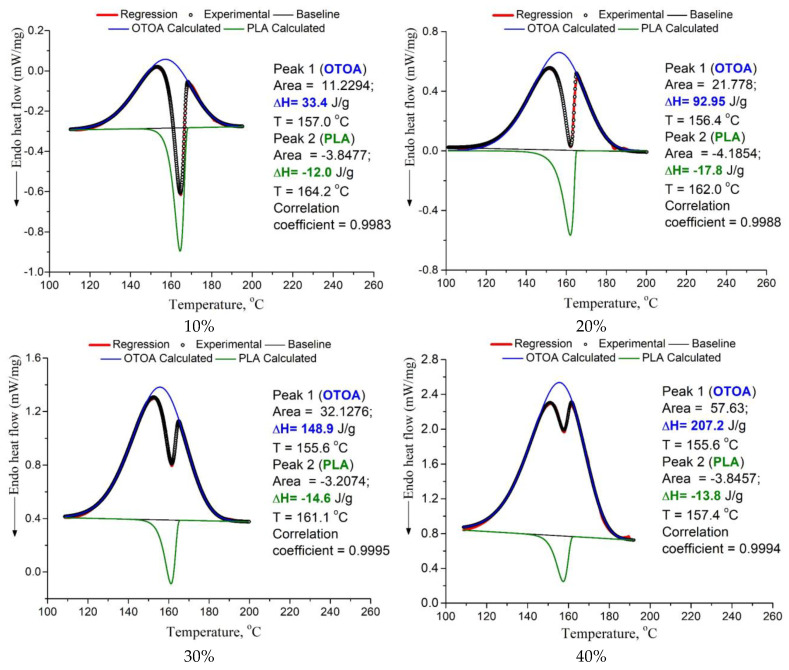
Deconvolution of the overlapping exo- and endothermic peaks in the DSC thermograms for PLA/PCL + OTOA films with 10–40% OTOA content.

In the DSC curves of PLA/PCL + OTOA films with more than 5% OTOA, a complex transition consisting of overlapping exo- and endothermic calorimetric peaks was observed in the temperature range of 140–180 °C (Figure 4b). The appearance of such a peak was associated with the introduction of OTOA into the PLA/PCL film. PLA melting corresponded to the endothermic peak, whereas the exothermic peak could be ascribed to the complex irreversible reaction of OTOA thermal destruction with the formation of C-OH groups [42]. Analysis of such complex calorimetric peaks was possible by deconvolution of the corresponding DSC curves using the well-established approaches [17,20], which made it possible to determine the PLA melting temperature (T_m_) and temperature of OTOA thermal degradation (T_d_), and estimate the melting enthalpy (ΔH_m_) and the degree of crystallinity for PLA/PCL + OTOA films with 10–40% OTOA content (Figure 6, Table 2). The degree of crystallinity (χ) values for the studied PLA/PCL films were obtained using Equation (1), taking into account the cold crystallization upon heating, and the corresponding values are given in Table 2.

The reference PLA/PCL film was characterized by a degree of crystallinity of 34.0%, as estimated by DSC, which is quite high for isothermally crystallized PLA films and is comparable with the χ value obtained previously for pure PLA film [17]. This could be explained by the high mobility of PLA chains in the solution providing the thermodynamic driving force required for the growth of PLA spherulites. In addition, cold-crystallization exothermic peaks were not observed in the DSC thermogram for the reference PLA/PCL film and PLA + OTOA film samples with low OTOA content (1 and 5% OTOA), being in agreement with the previously obtained results for PLA + OTOA films [17,18]. The absence of cold-crystallization peaks for PLA + OTOA samples with low OTOA content could be attributed to the slower crystallization kinetics and decreased nucleation density due to the addition of small amounts of OTOA [43].

Table 2 shows that OTOA introduction into PLA/PCL films led to the plasticizing effect, which is manifested in the decrease in the PLA melting temperature and decrease in the glass transition temperature (from 51.0 to 40.5 °C). The efficiency of the plasticizer in decreasing the T_g_ of PLA generally increases with OTOA content in the PLA/PCL film, being in accordance with the previously obtained results [17]. As can be seen from Table 2, both the melting temperature and the degree of crystallinity for the PLA/PCL films showed a downward trend with the increase in the OTOA content. The decrease in T_m_ could be attributed to the plasticizing action of OTOA, which was observed previously [17,18]. 

At the same time, the decrease in the degree of crystallinity for PLA/PCL films upon OTOA addition has a more complex character. Thus, after a significant decrease from 34.0% for the reference PLA/PCL film to 16.8% for the PLA/PCL + 10% OTOA film, crystallinity shows some increase for the PLA/PCL films with 20 and 30% of OTOA. The first part of this observed trend could be attributed to the intermolecular interaction between the PLA terminal –OH groups and OTOA molecules observed by FTIR, leading to the decrease in the mobility of PLA polymer chains. This could hinder PLA crystallization and lead to the decreased crystallinity of PLA/PCL films upon an increase in OTOA content. The observed increase in PLA/PCL film crystallinity upon a further increase in OTOA content could be accounted for by the dilution mechanism proposed previously [44]. When PLA and OTOA are mixed together in melt, OTOA molecules reduce the entropic barrier for nuclei formation, leading to a large number of spherulites forming in plasticized PLA. After nucleation, the crystal growth is dependent on the diffusion of PLA chains through the melt into the forming lamellae. OTOA molecules could decrease the local melt viscosity at the vicinity of the growing lamellae and additionally provide an increase in the lamellae lateral surface energy, leading to faster crystal growth. In order to efficiently enhance crystallinity, the optimum OTOA concentration has to be reached to trigger the dilution effect [44]. In our case, such a concentration is 20% OTOA content in the PLA/PCL film.

### 3.3. X-ray Diffraction Analysis

**Figure 7:**
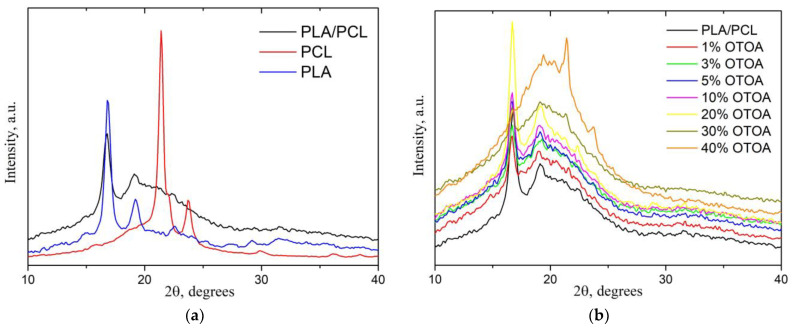
XRD patterns of PLA, PCL and PLA/PCL films (**a**); XRD patterns of PLA/PCL + OTOA film samples with 0–40% OTOA (**b**).

Figure 7 shows the X-ray diffraction patterns of pure PLA, PCL, PLA/PCL and PLA/PCL films loaded with different amounts of OTOA. The X-ray diffraction (XRD) pattern of the PLA/PCL blend film was compared with the patterns of pure PCL and PLA films to localize the characteristic diffraction peaks (Figure 7a). Three distinct diffraction peaks characteristic of the PLA crystalline α phase were visible in the XRD pattern of the pure PLA film at 2θ angles of 16.8°, 19.2° and 22.6° [45,46]. The XRD pattern for the PCL film showed strong diffraction peaks at 21.4° and 23.7° 2θ, which is consistent with the literature [47,48,49]. The PLA/PCL film showed main diffraction peaks corresponding to the PLA crystalline α phase at 16.8° and 19.2° 2θ and no visible diffraction peaks related to crystalline PCL (Figure 7a). This supports the previously made assumption that PCL in the PLA/PCL film is amorphous. As can be seen from Figure 7b, the reference PLA/PCL film and PLA/PCL + OTOA films with OTOA content up to 20% generally possessed the same crystalline structure. An increase in the mass fraction of OTOA did not lead to a significant change in the position of the diffraction peaks corresponding to the crystalline phase of PLA. Moreover, PLA/PCL film with 20% OTOA showed more intense XRD diffractions as compared to the reference PLA/PCL film. Similar behavior was observed for this film as was judged from the DSC data. This shows that the change in the degree of crystallinity for PLA/PCL films upon OTOA addition has a more complex character.

A further increase in the OTOA content above 30% led to an increase in the intensity of the peaks attributed to the PCL crystalline phase, whereas the crystallinity of the PLA phase was decreased. On the other hand, there was an increase in the contribution of the amorphous phase (amorphous halo), corresponding to the OTOA phase and/or amorphous regions of the semi-crystalline PLA/PCL matrix. The degree of crystallinity of PLA/PCL films was estimated from the XRD patterns using Equation (2) given above (see Materials and Methods), and the corresponding values are given in Table 3.

As the mass fraction of OTOA increased, a noticeable decrease in the crystallinity from 34.9% for the reference PLA/PCL film up to 18.1% for the PLA/PCL film with 40% OTOA was observed. There was also a noticeable increase in the crystallinity of the PLA/PCL film with 20% OTOA concentration. Such behavior of the system could be apparently associated the dilution mechanism described above [44], with the 20% OTOA content in the PLA/PCL film representing the optimum additive concentration to trigger this effect. For a film containing 40% OTOA, the crystallinity of the system dropped to 18.1%. In the PLA/PCL + 40% OTOA mixture, the intensity of PCL characteristic crystalline peaks increased and the intensity of PLA crystalline peaks was significantly reduced, which indicates PLA amorphization. The XRD data supported the DSC results that OTOA appears to promote crystallization of PCL in the PLA/PCL mixture.

The values of the degree of crystallinity for PLA/PCL + OTOA films (excluding films with 10 and 20% OTOA concentrations) estimated by XRD correlated quite well with the corresponding values obtained from DSC data. The nature of the observed discrepancies could be explained by the presence of liquid degradation products of OTOA in PLA/PCL compositions containing more than 10% OTOA at temperatures about 160 °C during the DSC scan. The heat capacity of OTOA degradation products differs from the heat capacity of high-molecular-weight PLA, which affects the heat transfer processes in the melt and, ultimately, the magnitude of the endothermic effect of PLA melting. As a result, in the DSC measurements of PLA/PCL compositions with high OTOA concentrations, underestimated values of the PLA degree of crystallinity could be obtained as compared to the XRD data (Table 2 and Table 3).

### 3.4. Morphology of PLA/PCL + OTOA Films

**Figure 8:**
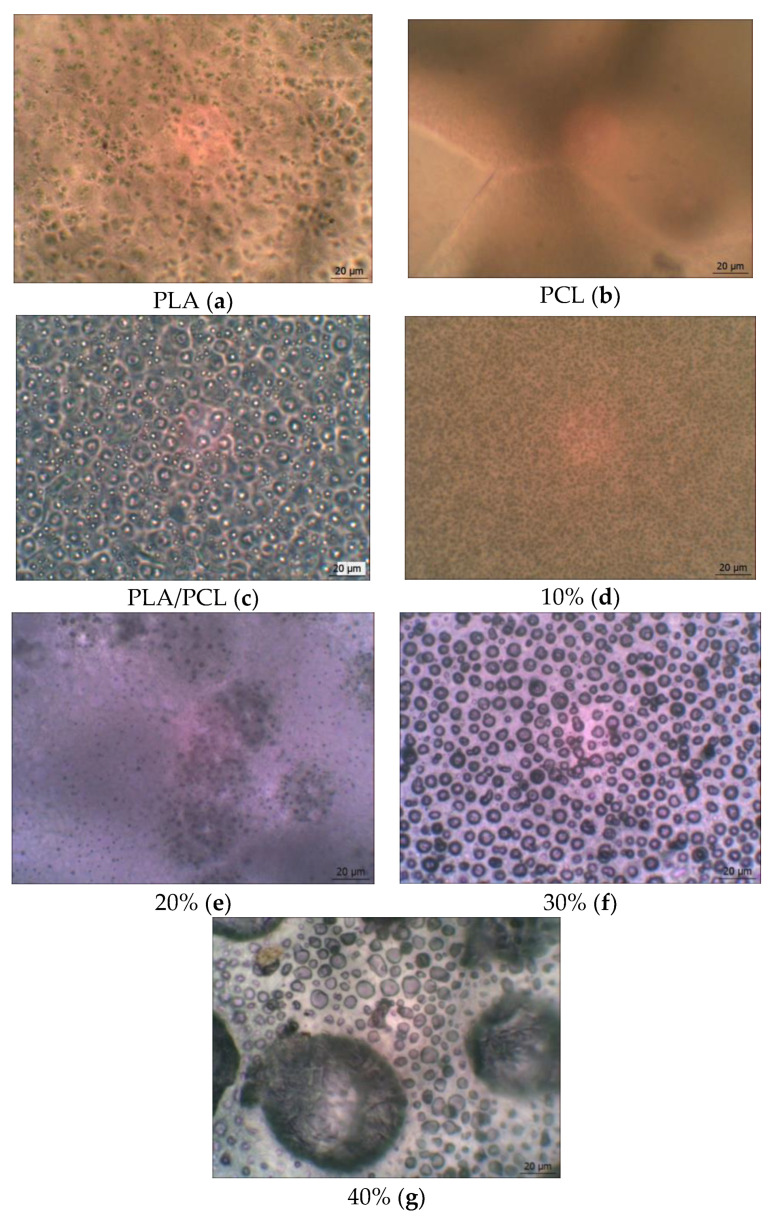
Microphotographs of the pristine PLA, PCL, PLA/PCL and PLA/PCL + OTOA film samples (10–40% OTOA), obtained with an optical microscope (**a**–**g**).

The introduction of OTOA significantly changed the appearance and morphology of PLA/PCL films, their structure and surface properties (Figure 8). The thickness of films with different OTOA concentrations did not change significantly and had average values of 116 ± 50 μm.

Like most polymer pairs, PLA and PCL are thermodynamically immiscible [50,51,52]. This results in the separation of macrophases during solvent evaporation and a reduction of the interfacial area between PLA and PCL in the film volume, leading to the formation of separate PCL domains inside the PLA matrix. This behavior and phase localization of PCL in the PLA matrix could influence the structure and morphology of PLA/PCL films upon OTOA addition, due to a possible difference in affinity between the additive (OTOA) and the two matrix components. In this case, the additive prefers to be dispersed in the component with higher affinity during mixing. With a reduced difference in the volumetric characteristics of polymer pairs, the filler will be located at the interface with a reduced interphase energy level. In the original PLA/PCL film, where the polymer ratio is 80/20, PCL is distributed in the PLA matrix in the form of spheres, which is consistent with previous reports [42,53]. However, when changing the mass fractions of the polymers due to the introduction of OTOA (see Table 1), a change in the distribution of polymer phases in the matrix was observed. In PLA/PCL + 40% OTOA film, the phase content was 48/12/40 and the OTOA fraction was greater than PCL. Therefore, phase inversion of PCL and OTOA was observed and OTOA pushed PCL to the interface with PLA. Thus, it can be seen that PCL interferes with the distribution of OTOA in the PLA matrix, which was established in the previous study [17]. By being located at the phase boundary, PCL prevents the fusion of discrete PLA domains, which in turn could decrease the PLA crystallinity. Moreover, the formation of such an interface PCL could enhance PCL-OTOA interactions und thus promote PCL crystallization, as was shown by XRD and DSC.

Morphological analysis showed that the structure of the films with an OTOA concentration above 20% was drastically changed. Specifically, an interface between the PLA/PCL matrix and OTOA was formed, thereby forming a capsule with the embedded OTOA. The observed morphology of PLA/PCL films with high OTOA content could be suitable for the potential application of developing materials as antibacterial agents.

### 3.5. Mechanical Properties of the Films

**Figure 9:**
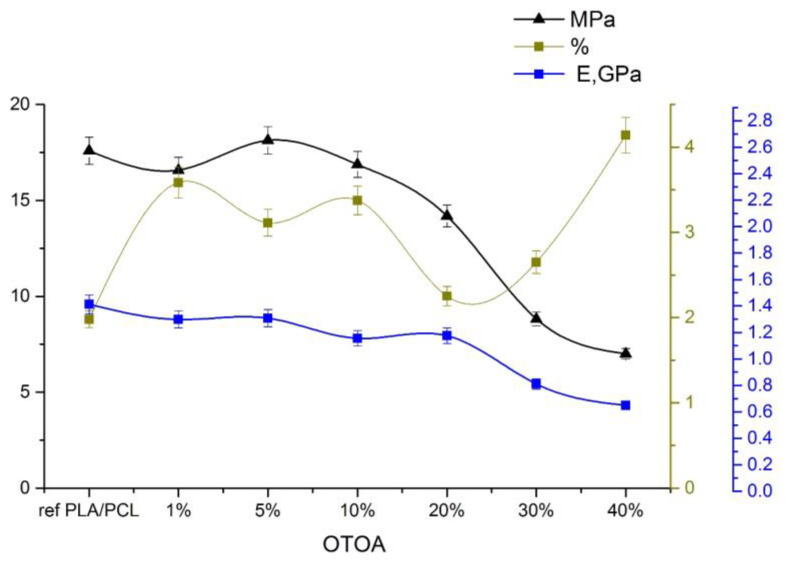
Elastic modulus (blue), tensile strength (black), and relative elongation at break (green) for reference PLA/PCL and PLA/PCL + OTOA (0–40%) film samples.

As the concentration of OTOA in the PLA/PCL film increases, the plastic deformation also increases [54]. The introduction of other components into the PLA matrix is usually associated with the desire to reduce the fragility of PLA materials and improve the elasticity of the resulting materials. Research data shows that the introduction of PCL into the matrix changes the mechanical properties of the material, reducing its strength and improving elasticity (Figure 9). When OTOA was introduced into the PLA/PCL mixture, the strength of the matrix decreased slightly to an OTOA concentration of 20%. However, already at 30% of the additive, it reached 8 MPa, and then at 40% OTOA the strength decreased to 5.6 MPa. For elastic modulus E, the decrease was not so significant—about 40%. This effect is probably associated with the reorganization of short side chains of macromolecules inside a multicomponent film.

The elasticity of the PLA/PCL film increased up to an OTOA concentration of 5%, then the behavior changed, which is apparently due to the inversion of PCL and its displacement to the interface between the PLA and OTOA phases. In the PLA/PCL + 20% OTOA film, the phase ratio was 64/16/20 (see Table 1), i.e., almost comparable amounts of PCL and OTOA, which, being redistributed in the PLA matrix, reduced its elasticity. The noticeably increased proportion of OTOA 30% and 40% in PLA/PCL mixtures and the already occurring phase inversion of OTOA and PCL increased the elasticity of the mixture to 4.5%. Amorphization of the material was observed. Our previous study [17,18] on the introduction of OTOA into a PLA matrix showed an increase in the size of spherulites with an increasing amount of additive. However, the introduction of an additional component of the PCL matrix, which itself is an excellent plasticizer, led to the absence of spherulites in the PLA/PCL blend films. The strength characteristics of the obtained films indicated a decrease in the strength and elastic modulus of the films.

### 3.6. Water Contact Angle, Water Sorption and Water Vapor Permeability of the Films

**Figure 10:**
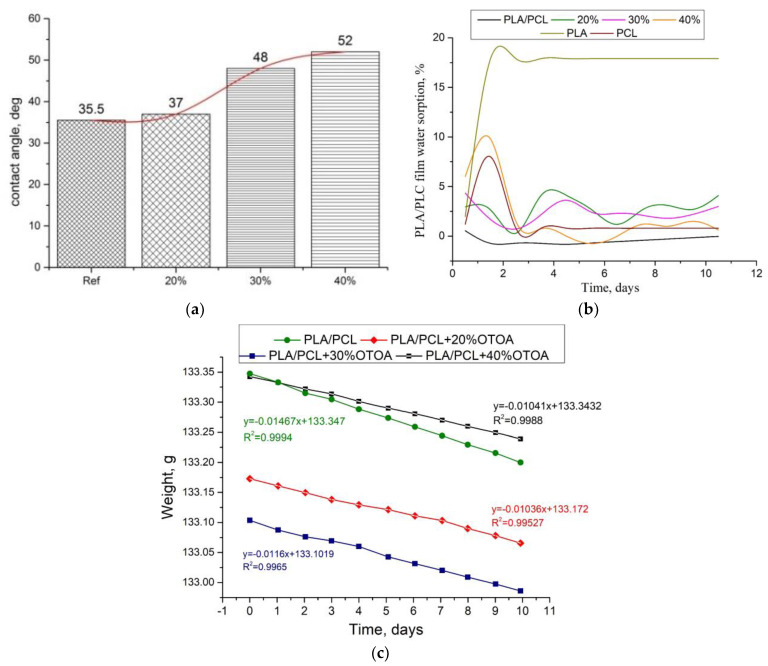
(**a**) Water contact angle for PLA/PCL films with variable OTOA content; (**b**) sorption capacity (Q) of PLA/PCL films with variable OTOA content; (**c**) weight loss versus time data from a water vapor permeability experiment.

To determine the hydrophilic–hydrophobic properties of the film surface, the water contact angle was measured for PLA/PCL films with varying OTOA content (Figure 10a). For the reference PLA/PCL film, good water wetting of the film surface was observed with a contact angle of 35.5° (the contact angle is noticeably less than 90°) [55]. When OTOA was added in an amount between 20% and 40%, the contact angle gradually increased to 52°, which indicates additional hydrophobization of the film surface. 

With an increasing amount of OTOA in the PLA/PCL matrix, a significant increase in the surface hydrophobicity of PLA/PCL + OTOA films was observed. The most pronounced hydrophobic properties were observed for the PLA/PCL film with 40% OTOA content. The study of film sorption characteristics showed that the reference PLA/PCL film already showed good hydrophobicity due to the presence of hydrophobic PCL in the film (Figure 10b) [56]. Sorption characteristics at room temperature do not appear for this film. The study was carried out at a temperature of 35 °C, since the intended possible applications of developed mixed PLA/PCL films assume their use in direct contact with human skin [57,58]. The structure of the OTOA molecule consists mainly of hydrophobic fragments and includes, along with low-polar ester groups, specific ozonide rings [59]. Only PLA/PCL film with 40% OTOA showed sorption characteristics similar to those of the PCL film. All other PLA/PCL + OTOA films exhibit sorption–desorption of moisture. Apparently, this is due to the distribution of fragments of OTOA molecules in the PLA/PLC matrix and the low crystallinity of the films. 

The mechanism for the penetration of water molecules through the developed films can be understood by the measurements of the vapor permeability (Figure 10c). The most widely used method for measuring vapor permeability of polymer films is the ASTM Standard Test Method E96, also known as the cup method. This method is based on calculating the WVP of a sample using gravimetry (see Equations (4)–(6) in Materials and Methods). In this method, a selected film sample covers the neck of the cup with the distilled water inside it, which creates a certain water vapor pressure depending on the temperature. The sealed cup is placed in a sealed chamber or desiccator with silica gel and stored at a constant temperature. The partial pressure gradient between both sides of the film due to different relative humidities creates a driving force that promotes the flow of water through the film. Since the cup contains distilled water, the flow of water vapor through the film causes the weight of the cup to decrease. Under dynamic equilibrium conditions, the weight loss of the cup will be constant, and the permeability of the film at a given temperature will be calculated from it. To calculate the permeability parameters, it is necessary to take into account the average film thickness of 8.7 × 10^−5^ (m) for the PLA/PCL + 40% OTOA sample and 12.8 × 10^−5^ (m) for the reference PLA/PCL film, ΔP inside and outside the cup at 25 °C (3169 Pa) and the neck area of 1.766 × 10^−4^ (m^2^), which is equal to the area of the film through which water molecules penetrate. Absolute slope values are used since we are only interested in the change in weight over time. WVTR is calculated by dividing the slope value by the neck area. From the WVTR value, the water vapor permeance for each sample is calculated by dividing the WVTR by ΔP. Finally, WVP is calculated by multiplying the permeance by the thickness of the measured sample (see Table 4).

The effect of the crystallinity of PLA/PCL films on water vapor permeability remains controversial at this time. In the conventional concept of a semicrystalline polymer material, an increase in crystallinity usually results in a decrease in water vapor sorption, since there is less material available to absorb water [51,60]. However, it is generally accepted that the water vapor sorption of PLA increases with crystallization. This is due to the decompression of the amorphous phase during crystallization, which is associated with the formation of RAF (hard amorphous fraction of PLA), where there is excess free volume for gas absorption [61,62]. In our case, the films have bulk hydrophobic, uniformly distributed PCL/OTOA phases that prevent the penetration of water vapor. Most biopolymer and biocompatible polymer films are highly hydrophilic due to the presence of a large number of hydrogen bonds, which causes poor barriers to water vapor [23]. The presence of OTOA in PLA/PCL blend films, which is a strong hydrophobic agent, reduces the WVP value by 47% as compared to the reference PLA/PCL films (Table 4).

The obtained results reveal the good vapor permeability of the developed PLA/PCL film materials, which at the same time are able to create a moist environment on the surface of the wound by reducing the loss of water vapor from open tissues, for example in shallow wounds. The films do not absorb water and, as can be seen from the contact angle measurements, begin to repel it. A study of the antibacterial activity of the resulting materials can determine how effective these mixed films are as a barrier against external pollution and bacteria. 

### 3.7. Antibacterial Activity of the Developped PLA/PCL + OTOA Films

To measure the antimicrobial activity of OTOA, we used the paper disk method with several bacterial strains such as *Pseudomonas aeruginosa*, *Raoultella terrigena* (*Klebsiella terrigena*) and *Agrobacterium tumefaciens*. The choice of bacteria was determined by their antibacterial resistance [63,64,65,66]. *Raoultella terrigena* (*Klebsiella terrigena*) is an opportunistic pathogen. *R. terrigena* infection can damage different organs such as lungs, as well as wounds, and promote general septic infection, especially in patients with chronic diseases. The mortality of this infection is about 44%, and in 38.6% of cases *R. terrigena* has a multidrug resistance antibiotic sensitivity profile [67]. 

More strains of pathogens have become resistant to antibiotics, and some have become resistant to multiple antibiotics and chemotherapy drugs—the phenomenon of multidrug resistance (MRD) [67]. Some of the strains have become resistant to almost all commonly available antimicrobial agents. In that sense, studies on the possible use of OTOA in application therapy (wound dressings) is of great importance, since OTOA has proven to be a powerful antibacterial agent [42,68,69].

**Figure 11:**
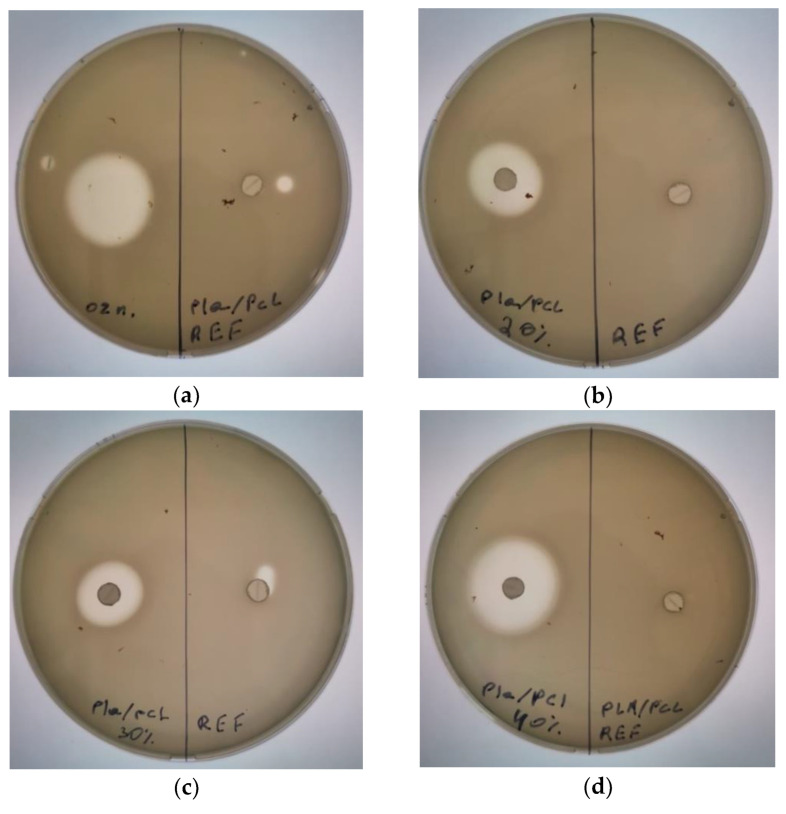
Comparison of antibacterial activities on *Pseudomonas aeruginosa* for: ref PLA/PCL film and the 100% OTOA (**a**); ref PLA/PCL film and the PLA/PCL films with 20% (**b**), 30% (**c**) and 40% (**d**) OTOA, respectively.

In Figure 11, one can see the results of antibacterial activity tests for PLA/PCL films on *Pseudomonas aeruginosa*. In Figure 11a, one can see the clear zone resulting from the application of pure 100% OTOA (the width of the clear zone is shown in the table below). Figure 11 also shows antibacterial activity on *Pseudomonas aeruginosa* for PLA/PCL + OTOA blend films with OTOA content from 20 to 40%. As can be seen, clear zones were observed for all PLA/PCL + OTOA samples, whereas the absence of antibacterial activity is visible for the reference PLA/PCL film. Similarly, antibacterial activity was shown using the paper disk method for other bacterial strains—*Raoultella terrigena* (*Klebsiella terrigena*) and *Agrobacterium tumefaciens*. The reference PLA/PCL films also did not show any activity against these bacteria strains. The observed point in Figure 11a at the Ref. part is the result of an accidental drop of OTOA on the Petri dish; in Figure 11c, the small clear zone is the result of a mechanical shift of the disk.

As can be seen from Table 5, the lysis zone generally increased with an increase in the OTOA content in the PLA/PCL films, and for the film with 40% OTOA, the effect was comparable with the antibacterial effect of pure OTOA. The antibacterial effect persisted for a long time, since the quantitative content of OTOA in the film was high, and the lysis zones remained free from bacterial growth. The obtained results showed that PLA/PCL films loaded with OTOA could be promising materials with high antibacterial activity for various bacteria strains.

The results obtained in this study indicate that it is possible to control a number of properties of blended PLA/PCL + OTOA films by changing the concentration of OTOA. These properties include the thermodynamic and mechanical properties of the films, morphology and vapor permeability, as well as the antibacterial activity of the films. Thus, a PLA/PCL film containing 40% OTOA is characterized by a combination of high antibacterial activity comparable to pure OTOA, reduced water permeability and additionally increased film hydrophobicity. In addition, PLA/PCL + 40% OTOA film showed a significant loss of tensile strength, which was accompanied with increased elasticity (relative elongation up to 4.5%). For PLA/PCL films, increasing elasticity while maintaining strength is important, because it expands the range of potential applications of the films. FT-IR spectroscopy revealed the interaction between the polar groups of the blended PLA/PCL matrix and the ozonide rings of OTOA, which supports the plasticizing rope of OTOA in the PLA/PCL compositions. DSC, XRD and mechanical tests showed that OTOA introduction led to complex changes in the thermal, structural and mechanical properties of the PLA/PCL + OTOA films depending on the OTOA content. The developed PLA/PCL films with various OTOA concentrations show a strong antibacterial effect on different bacterial strains with multidrug resistance behavior and could be regarded as promising materials for various biomedical applications.

## 4. Conclusions

In this paper, the physicochemical, thermal, mechanical, as well as antibacterial and morphological properties of PLA/PCL films were investigated after the addition of different concentrations of glycero-(9,10-trioxolane)-trioleate.

FTIR spectroscopy revealed interactions between the ozonide groups of OTOA and the PLA/PCL matrix. DSC and XRD data indicate a decrease in the degree of crystallinity of PLA/PCL + OTOA films relative to an increase in the OTOA content in the film. The change in the thermodynamic and mechanical properties of the blended films indicates an increase in the segmental mobility of the PLA/PCL + OTOA mixture and the influence of OTOA as a plasticizer on the system.

Also, the addition of OTOA gradually increases the contact angle to 52°, which indicates additional significant hydrophobization of the surface of the PLA/PCL + OTOA film.

Morphological analysis showed that the structure of the films with an OTOA content above 20% drastically changed, namely, PCL formed an interface between the matrix and OTOA, thereby forming a capsule with an antibacterial agent inside. The moisture permeability of the resulting PLA/PCL + OTOA films decreased due to the formation of uniformly distributed hydrophobic amorphous zones that prevent moisture penetration. These zones also affect the strength characteristics of the films: strength decreases to 5.6 MPa, elastic modulus E by 40%. The behavior of film elasticity is associated with the redistribution of amorphous regions in the matrix. Additionally, PLA/PCL + OTOA films with 20, 30 and 40% of OTOA showed good antibacterial properties on *Pseudomonas aeruginosa*, *Raoultella terrigena* (*Klebsiella terrigena*) and *Agrobacterium tumefaciens*. The developed PLA/PCL + OTOA films could be used as packaging materials and wound dressings. By changing the concentration of introduced OTOA, it is possible to tune the necessary physico-chemical and antibacterial properties of the developed films for various applications. The presence of OTOA, as a strong hydrophobic agent in PLA/PCL blend films, reduces the WVP value by 47% compared to PLA/PCL films.

The good vapor permeability of the resulting materials makes it possible to create a moist environment on the surface of the wound by reducing the loss of water vapor from open tissues, for example, in shallow wounds. The developed PLA/PCL + OTOA film materials could be used in various packaging and biomedical applications, with the possibility to tune physico-chemical properties of the films based on the requirements important for the specific application.

## Figures and Tables

**Table 1:** Percent concentrations and the mass fraction values of PLA/PCL + OTOA compositions.

Sample	Mass Percent Concentration (%)/Mass Fraction (α, β, γ) *(α* + *β* + *γ* = 1)					
	PLA	α	PCL	β	OTOA	γ
PLA/PCL ref	80	0.8	20	0.2	-	-
PLA/PCL + 1%	79.2	0.792	19.8	0.198	1	0.01
PLA/PCL + 5%	76	0.76	19	0.19	5	0.05
PLA/PCL + 10%	72	0.72	18	0.18	10	0.1
PLA/PCL + 20%	64	0.64	16	0.16	20	0.2
PLA/PCL + 30%	56	0.56	14	0.14	30	0.3
PLA/PCL + 40%	48	0.48	12	0.12	40	0.4

**Table 2:** Thermodynamic and structural characteristics of PLA/PCL + OTOA films (0–40% OTOA) obtained using the DSC method.

Sample	T_g_ PLA (°C)	T_m_ PCL (°C)	T_m_ PLA (°C)	T_cc_ PLA (°C)	T_d_ OTOA (°C)	Δ*H_cc_* PLA (J/g)	Δ*H_m_* PLA (J/g)	Δ*H_d_* OTOA (J/g)	χ(DSC) PLA (%)
PLA/PCL	51.0	58.9	168.4	n/a	n/a	n/a	31.37	n/a	34.0
PLA/PCL + 1%	50.6	59.1	167.9	n/a	n/a	n/a	28.37	n/a	30.4
PLA/PCL + 5%	49.4	58.7	166.8	86.7	n/a	2.05	25.18	n/a	24.8
PLA/PCL + 10%	48.8	57.8	164.2 *	86.6	157.0 *	2.08	16.77 *	334.0 *	16.4
PLA/PCL + 20%	48.0	57.4	162.0 *	87.9	156.4 *	3.47	22.43 *	464.0 *	20.3
PLA/PCL + 30%	47.0	57.4	161.1 *	85.7	155.6 *	9.79	26.07 *	488.0 *	20.2
PLA/PCL + 40%	40.5	62.5 (56.7)	157.4 *	95.8	155.6 *	14.08	28.75 *	518.0 *	15.7

* T_m_, T_d_, Δ*H_m_* and Δ*H_d_* values were obtained as a result of deconvolution of overlapping calorimetric peaks (see below); T_g_ values were obtained from the DSC thermograms of the cooling of the PLA/PCL films after the first DSC scan. The Δ*H_m_*, Δ*H_cc_* and Δ*H_d_* values are provided, taking into account the mass fraction of the PLA and OTOA in the composition.

**Table 3:** Crystallinity of PLA/PCL + OTOA films (0–40%) obtained using the XRD method.

Sample	PLA/PCL	PLA/PCL + 1%	PLA/PCL + 5%	PLA/PCL + 10%	PLA/PCL + 20%	PLA/PCL + 30%	PLA/PCL + 40%
*χ* (XRD) (%)	34.9	28.8	24.7	25.2	34.6	21.2	18.1 *

* PCL peaks prevail in the XRD pattern.

**Table 4:** Moisture permeability of different PLA/PCL films determined using moisture permeable cup method.

Sample	WVTR [g/d × m^2^]	Permeance [g/d × m^2^ × Pa]	WVP [g/d × m × Pa]
PLA/PCL	84.08	0.0265	3.39 × 10^−6^
PLA/PCL + 20% ОТОА	60.8	0.019	2.5 × 10^−6^
PLA/PCL + 30% ОТОА	67.46	0.0212	2.3 × 10^−6^
PLA/PCL + 40% ОТОА	58.43	0.0184	1.6 × 10^−6^

**Table 5:** Antibacterial activities of pure OTOA, reference PLA/PCL film and PLA/PCL films with 20, 30 and 40% OTOA on *Pseudomonas aeruginosa*, *Raoultella terrigena* (*Klebsiella terrigena*) and *Agrobacterium tumefaciens*.

Sample	Size of Clear Zone (mm)		
Bacterial Strain	*Pseudomonas aeruginosa*	*Raoultella terrigena* *(Klebsiella terrigena)*	*Agrobacterium tumefaciens*
PLA/PCL Ref OTOA 0%	0.0 ± 0.0	0.0 ± 0.0	0.0 ± 0.0
OTOA 100%	26.2 ± 0.1	25.5 ± 0.1	25.0 ± 0.1
PLA/PCL + 20%	21.1± 0.2	20.0 ± 0.1	20.0 ± 0.2
PLA/PCL + 30%	20.1 ± 0.2	23.0 ± 0.1	21.0 ± 0.1
PLA/PCL + 40%	28.1± 0.2	24.5 ± 0.1	22.7 ± 0.3

## Data Availability

The data presented in this study are available on request from the corresponding author.

## References

[1] Imre B., Pukánszky B. (2015). Compatibilization in bio-based and biodegradable polymer blends. Eur. Polym. J..

[2] Broz M.E., Vanderhart D.L., Washburn N.R. (2003). Structure and mechanical properties of poly(d, l-lactic Acid)/poly(ε-caprolactone) blends. Biomaterials.

[3] Formela K., Zedler Ł., Hejna A., Tercjak A. (2018). Reactive extrusion of bio-based polymer blends and Composites—Current trends and future developments. Express Polym. Lett..

[4] Noroozi N., Schafer L.L., Hatzikiriakos S.G. (2012). Thermorheological properties of poly(ε-caprolactone)/polylactide blends. Polym Eng Sci..

[5] Patrício T., Glória A., Bártolo P. (2013). Mechanical and biological behavior of PCL and PCL/PLA scaffolds for tissue engineering applications. Chem. Eng. Trans..

[6] Kellomaki M., Törmälä P., Hollander A., Hatton P. (2003). Processing of Resorbable Poly-α-Hydroxy Acids for Use as Tissue-Engineering Scaffolds, Biopolymer Methods in Tissue Engineering.

[7] Faruk O., Bledzki A.K., Fink H.-P., Sain M. (2012). Biocomposites reinforced with natural fibers: 2000–2010. Prog Polym Sci..

[8] Krishnan S., Pandey P., Mohanty S., Nayak S.K. (2016). Toughening of polylactic acid: An overview of research progress. Polym.-Plast. Technol. Eng..

[9] Dell’Erba R., Groeninckx G., Maglio G., Malinconico M., Migliozzi A. (2001). Immiscible polymer blends of semicrystalline biocompatible components: Thermal properties and phase morphology analysis of PLLA/PCL blends. Polymer.

[10] Mohamed R.M., Yusoh K.A. (2015). Review on the Recent Research of Polycaprolactone (PCL). Adv. Mater. Res..

[11] Dwivedi R., Kumar S., Pandey R., Mahajan A., Nandana D., Katti D.S., Mehrotra D. (2020). Polycaprolactone as a Biomaterial for Bone Scaffolds: Review of Literature. J. Oral Biol. Craniofacial Res..

[12] Urquijo J., Guerrica-Echavarria G., Equiazabal J.I. (2015). Synergistic effects in mechanical properties of PLA/PCL blends with optimized composition, processing, and morphology. J. Appl. Polym. Sci..

[13] Vilay V., Mariatti M., Ahmad Z., Pasomsouk K., Todo M. (2009). Characterization of the mechanical and thermal properties and morphological behavior of biodegradable poly(L-lactide)/poly(ε-caprolactone) and poly(L-lactide)/poly(butylene succinate-co-L-lactate) polymeric blends. J. Appl. Polym. Sci..

[14] Ostafinska A., Fortelny I., Nevoralova M., Hodan J., Kredatusova J., Slouf M. (2015). Synergistic effects in mechanical properties of PLA/PCL blends with optimized composition, processing, and morphology. RSC Adv..

[15] Sood A., Granick M.S., Tomaselli N.L. (2014). Wound Dressings and Comparative Effectiveness Data. Adv. Wound Care (New Rochelle).

[16] Shyu A.P., Krakauer M. (2022). Bilayer Tegaderm™ Moisture Chamber. Ophthalmic Plast. Reconstr. Surg..

[17] Alexeeva O., Olkhov A., Konstantinova M., Podmasterev V., Tretyakov I., Petrova T., Koryagina O., Lomakin S., Siracusa V., Iordanskii A.L. (2022). Improvement of the Structure and Physicochemical Properties of Polylactic Acid Films by Addition of Glycero-(9,10-trioxolane)-Trialeate. Polymers.

[18] Olkhov A., Alexeeva O., Konstantinova M., Podmasterev V., Tyubaeva P., Borunova A., Siracusa V., Iordanskii A.L. (2021). Effect of Glycero-(9,10-trioxolane)-trialeate on the Physicochemical Properties of Non-Woven Polylactic Acid Fiber Materials. Polymers.

[19] Fischer E., Sterzel H., Wegner G. (1973). Investigation of the structure of solution grown crystals of lactide copolymers by means of chemical reactions. Colloid. Polym. Sci..

[20] Fraser R.D.B., Suzuki E. (1969). Resolution of overlapping bands. Functions for simulating band shapes. Analytical Chem..

[21] Opfermann J. (2000). Kinetic Analysis Using Multivariate Non-Linear Regression. J. Therm. Anal. Calorim..

[22] Hsieh Y.T., Nozaki S., Kido M., Kamitani K., Kojio K., Takahara A. (2020). Crystal polymorphism of polylactide and its composites by X-ray diffraction study. Polym. J..

[23] Dadras Chomachayi M., Jalali-arani A., Beltrán F.R., de la Orden M.U., Martínez Urreaga J. (2020). Biodegradable Nanocomposites Developed from PLA/PCL Blends and Silk Fibroin Nanoparticles: Study on the Microstructure, Thermal Behavior, Crystallinity and Performance. J. Polym. Environ..

[24] Debnath S., Madhusoothanan M. (2010). Water Absorbency of Jute—Polypropylene Blended Needle-punched Nonwoven. J. Ind. Text..

[25] Heib F., Schmitt M. (2016). Statistical Contact Angle Analyses with the High-Precision Drop Shape Analysis (HPDSA) Approach: Basic Principles and Applications. Coatings.

[26] https://www.astm.org/Standards/E96.htm.

[27] Turhan K., Şahbaz F. (2004). Water vapor permeability, tensile properties and solubility of methylcellulose-based edible films. J. Food Eng..

[28] Cazón P., Morales-Sanchez E., Velazquez G., Vázquez M. (2022). Measurement of the Water Vapor Permeability of Chitosan Films: A Laboratory Experiment on Food Packaging Materials. J. Chem. Educ..

[29] Wexler A. (1976). Vapor Pressure Formulation for Water in Range 0 to 100 °C. A Revision. J. Res. Natl. Bur. Stand. A Phys. Chem..

[30] Gennadios A., Weller C.L., Gooding C.H. (1994). Measurement Errors in Water-Vapor Permeability of Highly Permeable, Hydrophilic Edible Films. J. Food Eng..

[31] McHugh T.H., Avena-Bustillos F.L., Krochta J.M. (1993). Hydrophilic Edible Films: Modified Procedure for Water Vapor Permeability and Explanation of Thickness Effects. J. Food Sci..

[32] Murray P.R., Jo E., Turnidge B. (2007). Manual of Clinical Microbiology.

[33] Balouiri M., Sadiki M., Ibnsouda S.K. (2016). Methods for in vitro evaluating antimicrobial activity: A review. J. Pharm. Anal..

[34] Clinical and Laboratory Standards Institute (CLSI) (2012). Performance Standards for Antimicrobial Disk Susceptibility Tests; Approved Standard.

[35] Clinical and Laboratory Standards Institute (CLSI) (2009). Method for Antifungal Disk Diffusion Susceptibility Testing of Yeasts; Approved Guideline.

[36] Sundar N., Keerthana P., Kumar S.A., Ghosh S. (2020). Dual purpose, bio-based polylactic acid (PLA)-polycaprolactone (PCL) blends for coated abrasive and packaging industrial coating applications. J. Polym. Res..

[37] Ali S., Khatri Z., Oh K.W., Kim I.-S., Kim S.H. (2014). Preparation and Characterization of Hybrid Polycaprolactone/Cellulose Ultrafine Fibers via Electrospinning. Macromol. Res..

[38] Chee W.K., Ibrahim N.A., Zainuddin N., Rahman M.F.A., Chieng B.W. (2013). Impact Toughness and Ductility Enhancement of Biodegradable Poly(lactic acid)/Poly(#-caprolactone) Blends via Addition of Glycidyl Methacrylate. Adv. Mater. Sci. Eng..

[39] Arjmandi R., Hassan A., Eichhorn S.J., Haafiz M.K.M., Zakaria Z., Tanjung F. (2015). Enhanced ductility and tensile properties of hybrid montmorillonite/cellulose nanowhiskers reinforced polylactic acid nanocomposites. J. Mater. Sci..

[40] Kolstad J.J. (1996). Crystallization kinetics of poly(L-lactide co-meso-lactide). J. Appl. Pol. Sci..

[41] Ferri J.M., Fenollar O., Jorda-Vilaplana A., García-Sanoguera D., Balart R. (2016). Effect of miscibility on mechanical and thermal properties of poly(lactic acid)/polycaprolactone blends. Polym. Int..

[42] de Almeida Kogawa R.N., de Arruda E.J., Micheletti A.C., Cepa Matos M.d.F., Silva de Oliveira L.C., de Lima D.P., Pereira Carvalho N.C., de Oliveira P.D., de Castro Cunha M., Ojeda M. (2015). Synthesis, characterization, thermal behavior and biological activity of ozonides from vegetable oils. RSC Adv..

[43] Xiao H., Liu F., Jiang T., Yeh J.T. (2010). Kinetics and Crystal Structure of Isothermal Crystallization of Poly(lactic acid) Plasticized with Triphenyl Phosphate. J. Appl. Polym. Sci..

[44] Farid T., Herrera V.N., Kristiina O. (2018). Investigation of crystalline structure of plasticized poly (lactic acid)/ Banana nanofibers composites. IOP Conf. Series Mater. Sci. Eng..

[45] Liu Y., Jiang S., Yan W., He M., Qin J., Qin S., Yu J. (2020). Crystallization morphology regulation on enhancing heat resistance of polylactic acid. Polymers.

[46] Xiao H., Lu W., Yeh J.T. (2009). Effect of Plasticizer on the Crystallization Behavior of Poly (lactic acid). J. Appl. Polym. Sci..

[47] Sun H., Yu B., Han J., Kong J., Meng L., Zhu F. (2014). Microstructure, Thermal Properties and Rheological Behavior of PLA/PCL Blends for Melt-blown Nonwovens. Polymer.

[48] Solechan S., Suprihanto A., Widyanto S.A., Triyono J., Fitriyana D.F., Siregar J.P., Cionita T. (2022). Investigating the Effect of PCL Concentrations on the Characterization of PLA Polymeric Blends for Biomaterial Applications. Materials.

[49] Tenorio-Alfonso A., Vázquez Ramos E., Martínez I., Ambrosi M., Raudino M. (2023). Assessment of the structures contribution (crystalline and mesophases) and mechanical properties of polycaprolactone/pluronic blends. J. Mech. Behav. Biomed. Mater..

[50] Oztemur J., Yalcin-Enis I. (2021). Development of biodegradable webs of PLA/PCL blends prepared via electrospinning: Morphological, chemical, and thermal characterization. J. Biomed. Mater. Res. B Appl. Biomater..

[51] Bulatović V.O., Mandić V., Kučić Grgić D., Ivančić A. (2021). Biodegradable Polymer Blends Based on Thermoplastic Starch. J. Polym. Environ..

[52] Arvanitoyannis I., Nikolaou E., Yamamoto N. (1994). Novel biodegradable copolyamides based on adipic acid, isophorone diamine and α-amino acids. 3. Synthesis, study of properties and evaluation of their biodegradability for food packaging applications. Angew. Makromol. Chem..

[53] Bai H., Huang C., Xiu H., Gao Y., Zhang Q., Fu Q. (2013). Toughening of poly(l-lactide) with poly(ε-caprolactone): Combined effects of matrix crystallization and impact modifier particle size. Polymer.

[54] Jia S., Yu D., Zhu Y., Wang Z., Chen L., Fu L. (2017). Morphology, Crystallization and Thermal Behaviors of PLA-Based Composites: Wonderful Effects of Hybrid GO/PEG via Dynamic Impregnating. Polymers.

[55] Pankova Y.N., Shchegolikhin A.N., Iordanskii A.L., Zhulkina A.L., Ol’khov A.A., Zaikov G.E. (2010). The characterization of novel biodegradable blends based on polyhydroxybutyrate: The role of water transport. J. Mol. Liq..

[56] Zhou Z.-X., Chen Y.-R., Zhang J.-Y., Jiang D., Yuan F.-Z., Mao Z.-M., Yang F., Jiang W.-B., Wang X., Yu J.-K. (2020). Facile Strategy on Hydrophilic Modification of Poly(e-caprolactone) Scaffolds for Assisting Tissue Engineered Meniscus Constructs In Vitro. Front. Pharmacol..

[57] Lee S., Lee J., Choi K., Kim H., Park Y., Yoon J., Kim J.H., Ryu S. (2021). Polylactic Acid and Polycaprolactone Blended Cosmetic Microneedle for Transdermal Hispidin Delivery System. Appl. Sci..

[58] Sharma D., Saha D., Satapathy B.K. (2021). Structurally optimized suture resistant polylactic acid (PLA)/poly (ε-caprolactone) (PCL) blend based engineered nanofibrous mats. J. Mech. Behav. Biomed. Mater..

[59] Chieng B.W., Ibrahim N.A., Then Y.Y., Loo Y.Y. (2014). Epoxidized Vegetable Oils Plasticized Poly(lactic acid) Biocomposites: Mechanical, Thermal and Morphology Properties. Molecules.

[60] Lu Y., Chen Y.C., Zhang P.H. (2016). Preparation and Characterisation of Polylactic Acid (PLA)/Polycaprolactone (PCL) Composite Microfibre Membranes. Fibres Textiles East. Eur..

[61] Sangroniz A., Chaos A., Iriarte M., del Río J., Sarasua J., Etxeberria A. (2018). Influence of the Rigid Amorphous Fraction and Crystallinity on Polylactide Transport Properties. Macromolecules.

[62] Chen H., Cebe P. (2008). Vitrification and Devitrification of Rigid Amorphous Fraction of PET during Quasi-Isothermal Cooling and Heating. Macromolecules.

[63] Agyeman W.Y., Bisht A., Gopinath A., Cheema A.H., Chaludiya K., Khalid M., Nwosu M., Konka S., Khan S. (2022). A Systematic Review of Antibiotic Resistance Trends and Treatment Options for Hospital-Acquired Multidrug-Resistant Infections. Cureus.

[64] Lekhniuk N., Fesenko U., Pidhirnyi Y., Sękowska A., Korniychuk O., Konechnyi Y. (2021). *Raoultella terrigena*: Current state of knowledge, after two recently identified clinical cases in Eastern Europe. Clin. Case Rep..

[65] Adnan M., Khan S., Patel M., Alshammari E., Ashankyty I. (2013). *Agrobacterium*: A potent human pathogen. Rev. Med. Microbiol..

[66] Qin S., Xiao W., Zhou C., Pu Q., Deng X., Lan L., Liang H., Song X., Wu M. (2022). *Pseudomonas aeruginosa*: Pathogenesis, virulence factors, antibiotic resistance, interaction with host, technology advances and emerging therapeutics. Signal Transduct. Target. Ther..

[67] Catalano A., Iacopetta D., Ceramella J., Scumaci D., Giuzio F., Saturnino C., Aquaro S., Rosano C., Sinicropi M.S. (2022). Multidrug Resistance (MDR): A Widespread Phenomenon in Pharmacological Therapies. Molecules.

[68] Ugazio E., Tullio V., Binello A., Tagliapietra S., Dosio F. (2020). Ozonated Oils as Antimicrobial Systems in Topical Applications. Their Characterization, Current Applications, and Advances in Improved Delivery Techniques. Molecules.

[69] Moureu S., Violleau F., Haimoud-Lekhal D.A., Calmon A. (2015). Ozonation of sunflower oils: Impact of experimental conditions on the composition and the antibacterial activity of ozonized oils. Chem. Phys. Lip..

